# Low-osmolarity oral rehydration solution for childhood diarrhoea: A systematic review and meta-analysis

**DOI:** 10.7189/jogh.14.04166

**Published:** 2024-12-06

**Authors:** Mustafa Bin Ali Zubairi, Syeda Kanza Naqvi, Ayesha Arshad Ali, Ashraf Sharif, Rehana Abdus Salam, Zain Hasnain, Sajid Soofi, Shabina Ariff, Yasir Bin Nisar, Jai K Das

**Affiliations:** 1 Institute for Global Health and Development, Aga Khan University, Karachi, Pakistan; 2 University Library, Aga Khan University, Karachi, Pakistan; 3 The Daffodil Centre, The University of Sydney, a joint venture with Cancer Council NSW, Sydney, New South Wales, Australia; 4 Centre of Excellence in Women and Child Health, Aga Khan University, Karachi, Pakistan; 5 Department of Pediatrics & Child Health, Aga Khan University, Karachi, Pakistan; 6 Department of Maternal, Newborn, Child, and Adolescent Health and Ageing, World Health Organization, Geneva, Switzerland; 7 Division of Women and Child Health, Aga Khan University, Karachi, Pakistan

## Abstract

**Background:**

Oral rehydration solution (ORS) is crucial in the management of diarrhoea. Until the early 2000s, the standard formulation of glucose-based ORS with a total osmolarity of 311 mmol/L was being used for this purpose. However, due to concerns about sodium levels and cases of hypernatremia, a low-osmolarity ORS solution (LORS) with an osmolarity of 245mmol/L or less was developed to replace the standard ORS. With this systematic review, we aimed to assess the effectiveness of LORS compared to standard ORS for the treatment of acute and persistent diarrhoea.

**Methods:**

We comprehensively searched PubMed, CINAHL, the Cochrane Library, ClinicalTrials.gov, the World Health Organization (WHO) International Clinical Trials Registry Platform, and Scopus until 20 July 2023 for studies published after 1990 assessing the efficacy of LORS in acute and persistent diarrhoea in children under 10 years of age. Meta-analysis was conducted using the RevMan software. We performed log approximation for all the values for an outcome when studies reported arithmetic and geometric means per the Cochrane Handbook. We otherwise used the Cochrane Risk of Bias II tool to assess the risk of bias in individual studies, and assessed the quality of evidence using the Grading of Recommendations, Assessment, Development, and Evaluations approach. This review was commissioned by the WHO for revision of guidelines for childhood diarrhoea.

**Results:**

For the comparison of LORS to standard ORS in acute diarrhoea, our findings suggest that there was a significant decrease in the duration of diarrhoea (mean difference (MD) = −0.28; 95% confidence interval (CI) = −0.41, −0.15; moderate certainty of evidence), stool output (MD = −0.25; 95% CI = −0.35, −0.16; very low certainty of evidence), and ORS intake (MD = −0.18; 95% CI = −0.28, −0.07; moderate certainty of evidence) in patients receiving LORS. There was a comparable effect on the number of patients cured within five days, treatment failure, and frequency of unscheduled intravenous therapy (risk ratio (RR) = 0.77; 95% CI = 0.72, 9.38; low certainty of evidence). For persistent diarrhoea, there was a significant decrease in duration of diarrhoea (MD = −30.60; 95% CI = −48.95, −12.25), stool output (MD = −14.00; 95% CI = −26.63, −1.37), and ORS intake (MD = −21.40; 95% CI = −41.01, −1.79), while there was a comparable effect on the number of patients cured.

**Conclusion:**

Our findings suggest that LORS should continue to be recommended in children under the age of 10 years with acute watery or persistent diarrhoea and upholds the current WHO recommendations.

**Registration:**

PROSPERO: CRD42023438762.

Diarrhoea, a persistent global health concern, is defined as the passage of three or more loose or liquid stools per day or an increase in stool frequency [1]. This condition significantly impacts children under the age of five in low- and middle-income countries (LMICs) and is responsible for approximately 11% of annual child deaths in these regions [2–4]. The Sustainable Development Goals set by the United Nations Assembly in 2015 aim at a reduction in the all-cause mortality of children under the age of five years to as low as 12 per 1000 by the year 2030, across all countries [5].

Depending on severity and length, the condition can be classified as acute watery diarrhoea (lasting less than 14 days), acute blood in the stool (dysentery), persistent diarrhoea (lasting 14–28 days), and chronic diarrhoea (lasting for more than 28 days) [3–6]. Each classification is associated with its distinct aetiological factors and clinical implications. Acute watery diarrhoea is typically caused by gastrointestinal pathogens like viruses and bacteria meanwhile, dysentery is frequently linked to salmonella or shigella species [2–7].

Diarrhoea leads to excessive excretion of faecal water, resulting in the loss of vital bodily fluids. This condition can stem from increased intestinal secretions or reduced intestinal absorption, leading to complications such as dehydration, electrolyte imbalances, and disruptions in the body's acid-base equilibrium [8,9]. In the long term, it can also lead to issues like malnutrition, growth failure, and cognitive delay. As a response, the WHO and the United Nations Children's Fund have actively worked to implement measures to reduce diarrhoea-related morbidity and mortality [10]. Although there has been a decline in mortality, the incidence of diarrhoea has remained constant. In response to the life-threatening complications of diarrhoea, oral rehydration solution (ORS) was introduced in the 1970s, as a crucial development for areas with limited access to intravenous fluid therapy [8].

The 1980s saw a surge in the availability and use of ORS, with access increasing from just 5% in 1982 to an impressive 61% in 1988 among children under the age of five [11,12]. However, global ORS use rates have stagnated over the past four decades as ORS is mostly used in about a third of the patient population with diarrhoea [9–11]. In their review, Das et al. [13] found that limited access to health care facilities and lack of trained human resources in primary health setups deprives the poorest of the populations of simple yet effective interventions, such as ORS which has lifesaving potential. They also observed that community-based interventions led to a significant increase in treatment modalities such as zinc and ORS [13].

Until the early 2000s, the standard formulation of glucose-based ORS was based on WHO recommendations; this formulation had a total osmolarity of 90 mmol/L of sodium, 111 mmol/L of glucose, and 80 mmol/L of chloride. However, due to concerns about sodium levels and cases of hypernatremia, a low-osmolarity ORS solution (LORS) was developed as a replacement for the standard ORS, with an osmolarity of 245mmol/L or less, with 75 mmol/L of sodium, 75 mmol/L of glucose, and 60 mmol/L of chloride [14]. Literature indicates that children who receive LORS are less likely to require intravenous infusion compared to those receiving standard ORS. Furthermore, LORS has been found to reduce stool output and vomiting during diarrhoea, with no significant difference in hyponatremia rates [15].

Despite the proven benefits of LORS over ORS, it is essential to continually assess and update the evidence on their efficacy and safety to ensure the best outcomes for children with diarrhoea, especially as many previous systematic reviews have included studies conducted before 1990. This is problematic because the standard ORS formula before 1990 contained bicarbonate, which was later replaced by citrate. Therefore, newer reviews would have to focus on studies using the updated formulations to provide more accurate and relevant conclusions.

With this systematic review, we aimed to update the evidence and assess the impact of LORS on diarrhoea in children up to 10 years of age in terms of clinical cure, treatment failure, and duration of diarrhoea. The WHO commissioned this review in order to inform an update of current guidelines on diarrhoea management.

## METHODS

### Objective

Our aim was to assess the effectiveness of LORS (≥245 mmol/L) when compared to standard ORS (311 mmol/L) for the management of acute watery or persistent diarrhoea in children less than 10 years of age. The protocol for this review was registered with PROSPERO (CRD42023438762).

### Inclusion criteria

We included randomised controlled trials (RCTs) assessing the efficacy of LORS, characterised by an osmolarity of 245 mmol/L or less when compared to standard ORS (osmolarity 311 mmol/L) in children less than 10 years of age. We excluded non-randomised controlled trials, including quasi-experimental studies, controlled before-after, interrupted time series studies, observational studies, before-after studies with no control/comparison arm, case reports, case series, opinions, editorials, commentaries, conference abstracts, qualitative studies, grey literature, and reviews or systematic reviews.

### Outcomes

The following outcomes were assessed and defined according to the authors of the primary studies:

Primary outcomes:

− Clinical cure
− Treatment failure
− Duration of diarrhoea

Secondary outcomes:

− Stool output
− Unscheduled intravenous fluid infusion
− Rehydration ORS consumed

### Search strategy

We formulated the search strategy using the PICO methodology based on medical subject headings and keywords, but did not restrict it by the outcome to retain a broader search (Table S1–5 in the **Online Supplementary Document**). Studies from 1990 onwards and studies published in the English language were included. We conducted searches until 20 July 2023 in the following databases: PubMed, CINAHL, the Cochrane Library, ClinicalTrials.gov, the WHO International Clinical Trials Registry Platform, and Scopus. We also searched the reference list of all the included studies and relevant systematic reviews to retrieve any records missed by the initial search. Additionally, we put the title of each included study in Google Scholar and screened the first 50 results.

We exported the search results to EndNote 20 (Clarivate, London, UK), deduplicated them, and uploaded them on Covidence [16] for title, abstract, and full-text screening. At both the title/abstract and full-text screening stages, two review authors (KN, MZ) independently scanned and screened all records retrieved by the searches for relevance based on selection criteria (**Box 1**). Disagreements were resolved through discussion or by contacting a third review author (ZH or AA) when necessary.

Box 1 **Inclusion and exclusion criteria**

Inclusion criteria
− Low-, middle-, or high-income country
− Infants and children 0 months to 10 years (119 months) of age
− Type of intervention: low osmolarity ORS (osmolarity < 245 mmol/L)
− Comparison group: standard ORS (osmolarity 311 mmol/L)
− Relevant study designs: RCT (individual or cluster)
Exclusion criteria
− Recruitment of adults or animals.
− Irrelevant study designs: grey literature, case reports, case-control studies, case series, opinions, editorials, commentaries, letters, conference abstracts, and reviews or systematic reviews.
− Studies without a comparison group or showing a deviated composition from standard ORS.
− Studies which did not specify the composition of ORS
− Studies in languages other than English
− Studies conducted before the year 1990

### Data extraction and management

Two review authors (KN, MZ) independently extracted data from the included study onto a standardised data extraction form in Excel that had been piloted. Two independent authors (KN, MZ) assessed the quality of the included studies and disagreements were resolved by collaboratively re-evaluating the relevant studies, with a focus on identifying areas of agreement and resolving any remaining discrepancies through constructive discussion or involving a third author (ZH or AA). We used the Cochrane Risk of Bias II (RoB 2) tool to assess the methodological quality of RCTs (individual and cluster) and gave the overall risk of bias judgment for each study (low, high, some concerns) [17]. The tool for RCTs assessed trials on the following domains: randomisation process, deviations from the intended interventions, missing outcome data, measurement of the outcome, and selection of the reported result [17]. Emails were sent to the corresponding authors for any missing information, but we did not receive any unpublished data.

### Statistical analysis

We conducted a meta-analysis in RevMan, version 5.4.1. (The Cochrane Collaboration, London, UK). We presented dichotomous outcomes using risk ratios (RRs) and continuous outcomes using mean differences (MDs) along with 95% confidence intervals (CIs). We performed a random-effect analysis for all comparisons, using inverse-variance and Mantel-Haenszel methods to calculate the net estimate weights for continuous and categorical outcomes. For some of the outcomes in the comparison of acute diarrhoea, studies reported arithmetic means whereas others reported geometric means, hence, we log approximated all the values for that particular outcome using the formula proposed in the Cochrane Handbook [18].

We assessed statistical heterogeneity using τ^2^, *I*^2^, the significance of the χ^2^ test, and by visually inspecting forest plots. Where there was significant heterogeneity, we used random effect models. We further conducted subgroup analyses to explore potential sources for heterogeneity. For all continuous outcomes, we converted the units to a uniform scale. We calculated means and standard deviations (SDs) if the median and interquartile range (IQR)/CI were provided [18].

We conducted a subgroup analysis for any outcome with more than one study. The overall analysis included the data from the last reported follow-up of each included study. We also conducted subgroup analyses based on study region, categorising studies into either low-middle income or high-income countries based on the World Bank classification [19]. We also conducted a subgroup analysis based on the time of outcome reporting and the RoB 2 assessment.

### Sensitivity analysis

We performed sensitivity analysis on all outcomes to consider the impact of the high risk of bias related to sequence generation and/or allocation concealment. We did this by removing studies that had a high risk of bias or some concerns in more than one domain. We did not perform a sensitivity analysis in outcomes where all studies had a low risk of bias.

### Quality assessment (GRADE)

We conducted the Grading of Recommendations, Assessment, Development, and Evaluations (GRADE) for all the included outcomes using the GRADEpro software and generated evidence profile tables. We assessed the outcomes based on the risk of bias, inconsistency, indirectness, imprecision, and publication bias. The certainty of the evidence for each outcome was then rated as very low, low, moderate, or high [20–22] and presented alongside the reason for downgrading the evidence in the summary tables.

## RESULTS

**Figure 1:**
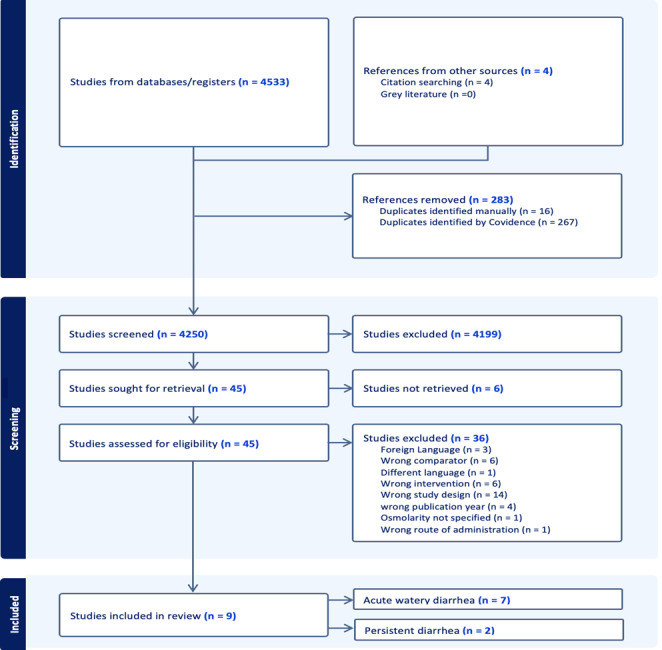
PRISMA flow diagram.

We identified 4250 de-duplicated records and removed 4205 ineligible ones at titles/abstracts screening. A total of 45 full texts were reviewed, with an addition of four studies from cross-referencing. We included nine RCTs with 1942 participants in our review (**Figure 1**).

### Study characteristics

Out of the total of nine RCTs, seven were conducted for acute diarrhoea and two for persistent diarrhoea (**Table 1**). In terms of sample size, the included RCTs involved between 61 and 676 participants across various age ranges: 0–2 months [29], 1–24 months [24,25,28], 3–24 months [23,30], 4–24 months [31], 6–48 months [27], and 3–59 months [26]. All studies were conducted in LMICs; three studies were conducted in Bangladesh [28,29,31], three in India [23,26,27], and two in Egypt [25,31]. One study was a multi-country study and included Brazil, India, Mexico, and Peru [24]. The osmolarity of LORS ranged from 210 mmol/L to 245 mmol/L. All studies were conducted in tertiary care settings.

**Table 1:** Study characteristics

Authors, year (reference)	Study design	Country	Setting	Type of diarrhoea	Osmolarity of low osmolar ORS (mmol/l)	Age range (in months)	Outcomes
El-Mougi et al., 1994 [23]	RCT	Egypt	Tertiary	Acute	210	3–24	Primary: duration of diarrhoea. Secondary: stool output, ORS intake
1995 [24]	RCT	Multi-country	Tertiary	Acute	224	1–24	Primary: duration of diarrhoea. Secondary: stool output, ORS Intake, unscheduled intravenous therapy
Santosham et al.,1996 [25]	RCT	Egypt	Tertiary	Acute	245	1–24	Primary: duration of diarrhoea, treatment failures. Secondary: stool output, ORS intake
Alam et al., 2000 [26]	RCT	India	Tertiary	Acute	245	3–59	Primary: duration of diarrhoea. Secondary: stool output, ORS intake, unscheduled intravenous therapy
Dutta et al, 2001 [27]	RCT	India	Tertiary	Acute	224	6–48	Primary: duration of diarrhoea, patients cured. Secondary: stool output, ORS intake
CHOICE, 2001 [28]	RCT	Bangladesh	Tertiary	Acute	245	1–24	Primary: duration of diarrhoea. Secondary: stool output, ORS intake, unscheduled intravenous therapy
Khan et al., 2005 [29]	RCT	Bangladesh	Tertiary	Acute	245	0–2	Primary: patients cured. Secondary: stool output, ORS intake, unscheduled intravenous therapy
Dutta et al., 2000 [30]	RCT	India	Tertiary	Persistent	224	3–24	Primary: patients cured, duration of diarrhoea. Secondary: Stool output, ORS intake
Sarker et al., 2001 [31]	RCT	Bangladesh	Tertiary	Persistent	208	4–24	Secondary: ORS intake

ORS – oral rehydration solution, RCT – randomised controlled trial

### Acute diarrhoea

#### Primary outcomes

**Figure 2:**
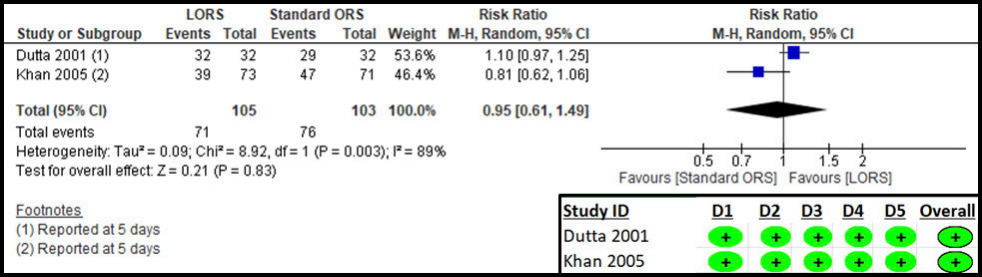
Forest plot and RoB 2 assessment for the number of patients cured within five days.

Two studies provided outcomes regarding the number of patients cured within five days [27,29]. The RoB 2 analysis showed a low risk of bias in all domains (**Figure 2**). The results suggest that there was no significant difference in cure rates between the LORS and standard ORS groups (n = 208; RR = 0.95; 95% CI = 0.61, 1.49; low certainty of evidence) (**Figure 2**, **Table 2**).

**Table 2:** GRADE for acute watery diarrhoea

Certainty assessment								Number of patients, n/N (%)		Effect			
**Outcome**	**Study design**		**Risk of bias**	**Inconsistency**	**Indirectness**	**Imprecision**	**Other considerations**	**LORS**	**WHO ORS**	**Indirectness, RR (95% CI)**	**AR per 1000 population (95% CI)**	**Certainty**	**Importance**
Patients cured at day 5(two studies)		RCT	Not serious	Not serious*	Not serious	Very serious†	None	71/105 (67.6)	76/103 (73.8)	0.95 (0.61, 1.49)	37 (288,362)	Low	Critical
Treatment failure (one study)		RCT	Not serious	Not serious	Not serious	Very serious‡	None	1/94 (1.1)	8/96 (8.3)	0.13 (0.02, 1.00)	73 (82, 0)	Low	Critical
Duration of diarrhoea in hours (six studies)		RCT	Not serious§	Serious*	Not serious	Not serious	None	784	765		MD = 0.29 h (0.42, 0.16)	Moderate	Critical
Stool output, g/kg (six studies)		RCT	Serious║	Very serious*	Not serious	Not serious	None	773	759		MD = 0.25 g/kg (0.35, 0.16)	Very low	Important
Stool output at rehydration phase, g/kg (one study)		RCT	Not serious	Not serious	Not serious	Serious**	None	94	96		MD 0.31 g/kg (0.35, 0.27)	Moderate	Important
Stool output at 24 h, g/kg (six studies)		RCT	Serious║	Serious††	Not serious	Serious¶	None	773	759		MD = 0.23 g/kg (0.3, 0.16)	Very low	Important
Stool output at 72 h, g/kg (one study)		RCT	Not serious	Not serious	Not serious	Very serious**	None	32	32		MD = 1.33 g/kg (1.63, 1.03)	Low	Important
Stool output at last follow-up, g/kg (four studies)		RCT	Not serious	Not serious	Not serious	Serious¶	None	667	655		MD = 0.24 g/kg (0.37, 0.1)	Moderate	Important
Frequency of unscheduled intravenous therapy (three studies)		RCT	Serious‡‡	Not serious	Not serious	Serious†	None	75/635 (11.8)	96/623 (15.4)	0.77 (0.58, 1.02)	35 (65, 3)	Low	Important
ORS intake, ml/kg (six studies)		RCT	Serious§§	Not serious*	Not serious	Not serious	None	769	754		MD 0.18 (0.28, 0.07)	Moderate	Important

AR – absolute risk, CI – confidence interval, MD – mean difference, ORS – oral rehydration solution, RCT – randomised controlled trial, RR – risk ratio, WHO – World Health Organization

*High heterogeneity, unchanged on subgroup analysis, CIs are overlapping.

†Confidence intervals are crossing null values.

‡Small sample size, CI approaching null.

§One study shows an overall high risk of bias, and three studies show some concerns.

¶Small sample size.

║One study shows an overall high risk of bias, and two studies show some concerns.

**Small sample size, only one study.

††*I*^2^ = 92%.

‡‡Two studies show some concerns.

§§One study has an overall high risk of bias while 3 studies show some concerns.

**Figure 3:**
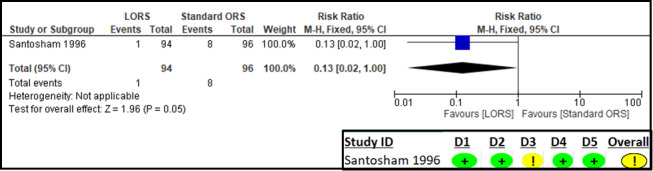
Forest plot and RoB 2 assessment for treatment failure.

Only one study reported on treatment failure [25] defining it as the ‘persistence of clinical signs of dehydration’ or ‘failure to maintain positive fluid balance for more than 12 hours after oral rehydration therapy’. The RoB 2 assessment suggested some concerns in the domain of missing outcome data (**Figure 3**). The results suggest no significant difference in treatment failure between the LORS and standard ORS groups (n = 190; RR = 0.13; 95% CI = 0.02, 1.00; low certainty of evidence) (**Figure 3**, **Table 2**).

**Figure 4:**
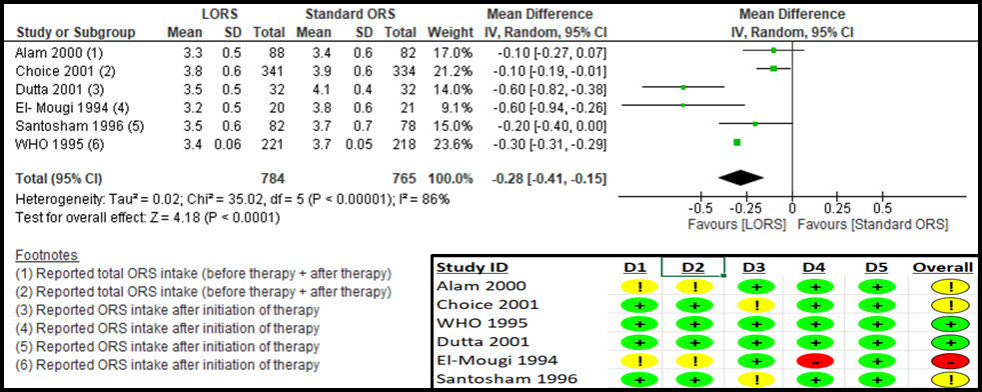
Forest plot and RoB 2 assessment for mean log-approximated duration of diarrhoea (h).

Six studies reported on the mean overall duration of diarrhoea (hours) [24–28,30] (Table S6 in the **Online Supplementary Document**). The RoB 2 assessment suggested some concerns for the randomisation process and deviations from intended interventions in one study [26], some concerns for missing outcome data in two studies [25,28], and some concerns in the randomisation process and deviations from intended interventions and a high risk of bias for the measurement of the outcome in one study [30] (**Figure 4**). The log-approximated results suggest there was a significant reduction in the overall duration of diarrhoea when the LORS group was compared to the standard ORS group (n = 1549; MD = −0.28, 95% CI = −0.41, −0.15; moderate certainty of evidence) (**Figure 4**, **Table 3**). The sensitivity analysis suggested no different results in the two studies [26,30] were removed due to concerns of risk of bias (Table S9 in the **Online Supplementary Document**). The subgroup analysis suggested a significant reduction for both LMICs (MD = −0.31, 95% CI = −0.51, −0.11) and mixed (LMIC/high-income country (HIC)) settings (MD = −0.30; 95% CI = −0.31, −0.29) (Figures S2–4 in the **Online Supplementary Document**).

**Table 3:** GRADE for persistent diarrhoea

Certainty assessment							Number of patients, n/N (%)		Effect			
**Outcome**	**Study design**	**Risk of bias**	**Inconsistency**	**Indirectness**	**Imprecision**	**Other considerations**	**LORS**	**WHO ORS**	**RR (95% CI)**	**AR per 1000 population (95% CI)**	**Certainty**	**Importance**
Patients cured (two studies)	RCT	Not serious	Not serious	Not serious	Very serious†	None	48/63 (76.2)	49/69 (71.0)	1.08 (0.91, 1.28)	57 (64, 199)	Low	Critical
Duration of diarrhoea (one study)	RCT	Not serious	Not serious	Not serious	Very serious*	None	33	37	-	MD = 30.6 (48.95, 12.25)	Low	Critical
Stool output (one study)	RCT	Not serious	Not serious	Not serious	Very serious*	None	33	37	-	MD = 14 (26.63, 1.37)	Low	Critical
ORS intake (one study)	RCT	Not serious	Not serious	Not serious	Very serious*	None	33	37	-	MD = 21.4 (41.01, 1.79)	Low	Important

AR – absolute risk, CI – confidence interval, LORS – low-osmolarity ORS solution, ORS – oral rehydration solution, MD – mean difference, ORS – oral rehydration solution, RCT – randomised controlled trial, RR – risk ratio

*Confidence intervals are crossing null values, small sample size.

†Small sample size, CI approaching null, only one study.

#### Secondary outcomes

**Figure 5:**
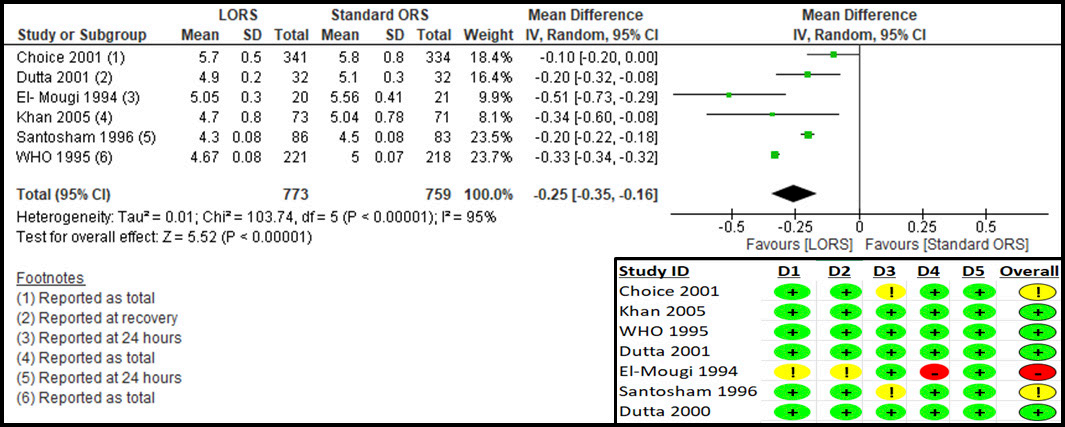
Forest plot and RoB 2 assessment for mean log-approximated stool output (g/kg).

Six studies reported on the mean overall stool output (g/kg) [24,25,27–30] (Table S7 in the **Online Supplementary Document**). The RoB 2 assessment suggested some concerns for missing outcome data for two studies [25,28], and some concerns for the randomisation process and deviations from intended interventions and a high risk of bias for the measurement of the outcomes for one study [30] (**Figure 5**). The log-approximated results suggest a significant reduction in overall stool output in the LORS group compared to the standard ORS group (n = 1532; MD = −0.25; 95% CI = −0.35, −0.16; very low certainty of evidence) (**Figure 5**, **Table 3**). The sensitivity analysis after removing the high-risk study [30] suggested no significant difference from the original analysis (Table S9 in **the** **Online Supplementary Document**). The results for the subgroup based on reporting time showed a significant reduction in stool output with LORS, during the ‘rehydration phase’ (MD = −0.31; 95% CI = −0.35, −0.27), at ‘24 hours’ (MD = −0.23; 95% CI = −0.30, −0.16), ‘72 hours’ (MD = -1.33; 95% CI = −1.63, 1.03), and ‘end of follow-up’ (MD = −0.24; 95% CI = −0.37, −0.10) (Annex 2, Table A5 in the **Online Supplementary Document**). Subgroups according to the study region suggested significant reduction for LMIC (MD = −0.22; 95% CI = −0.31, −0.13) and mixed (LMIC/HIC) (MD = −0.33; 95% CI = −0.34, −0.32) (Figures S5–7 in the **Online Supplementary Document**).

**Figure 6:**
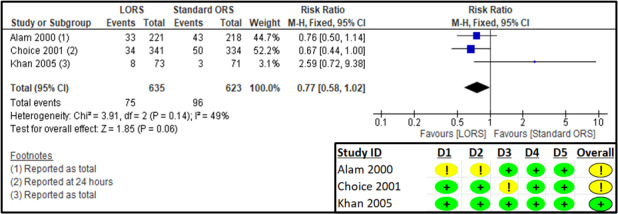
Forest plot and RoB 2 assessment for frequency of unscheduled intravenous fluid therapy.

Three studies reported on the overall frequency of unscheduled intravenous therapy [26,28,29]. The RoB 2 assessment suggested some concerns for the randomisation process and deviation from intended interventions in one study [26] and some concerns for missing outcome data for another [28] (**Figure 6**).

The results suggest no significant difference in the frequency of unscheduled intravenous therapy between the LORS and standard ORS groups (n = 1258; RR = 0.77; 95% CI = 0.72, 9.38; low certainty of evidence) (**Figure 6**; **Table 2**).

The sensitivity analysis after removing one study [26] showed no significant difference from the original analysis (Table S9 in the **Online Supplementary Document**). The results for the subgroup analysis based on reporting time suggested no significant reduction in the frequency of IV fluids with LORS, at both ‘end of follow-up’ (RR = 0.88; 95% CI = 0.60, 1.29) and at ‘24 hours’ (RR = 0.67; 95% CI = 0.44, 1.00) (Figures S8–9 in the **Online Supplementary Document**).

**Figure 7:**
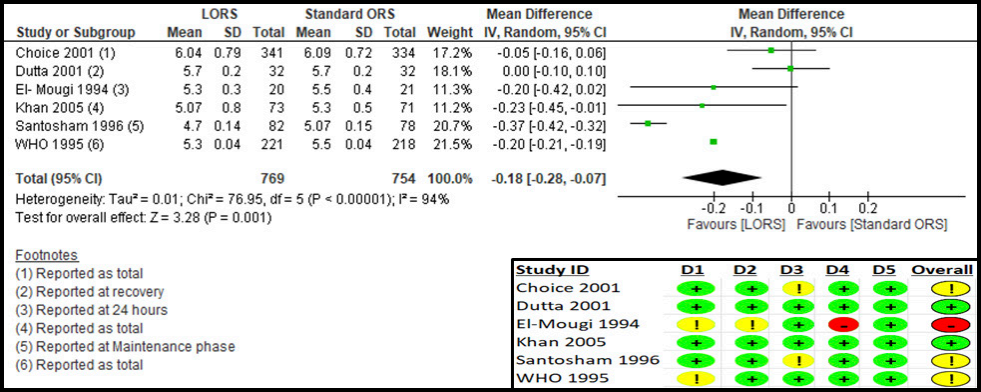
Forest plot and RoB 2 assessment for mean log-approximated ORS intake (ml/kg).

Six studies reported on the mean overall ORS intake (ml/kg) [24,25,27–30] (Table S8 in the **Online Supplementary Document**). The RoB 2 assessment suggested some concerns for missing outcome data in two studies [25,28], some concerns in the randomisation process and deviations from intended interventions and a high risk of bias for measurement of the outcome in one study [30], and some concerns for the randomisation process in one study [24] (**Figure 7**).

The log-approximated results suggest a significant reduction in ORS intake in the LORS group compared to the standard ORS group (n = 1532; MD = −0.18; 95% CI = −0.28, −0.07; moderate certainty of evidence) (**Figure 7**, **Table 3**). The sensitivity analysis after removing the high-risk study [30] showed no significant difference in ORS intake between the two treatment groups (Table S9 in the **Online Supplementary Document**). The results for the subgroup analysis based on reporting time suggested a significant reduction in ORS intake in the LORS group, during ‘the maintenance phase’ (MD = −0.37; 95% CI = −0.42, −0.32) and at ‘24 hours’ (MD = −0.25; 95% CI = −0.36, −0.13), while there was no significant difference during ‘the rehydration phase’ (MD = 0.00; 95% CI = −0.01, 0.01) and at ‘end of follow-up’ (MD = −0.11; 95% CI = −0.24, 0.01) (Table S5 in Annex 2 of the **Online Supplementary Document**). Subgroup analysis according to the study region suggested decreased stool output for LMIC (MD = −0.17; 95% CI = −0.36, −0.03) and mixed (LMIC/HIC) settings (MD = −0.20; 95% CI = −0.21, −0.19) (Figures S10–12 in the **Online Supplementary Document**).

### Persistent diarrhoea

#### Primary outcomes

**Figure 8:**
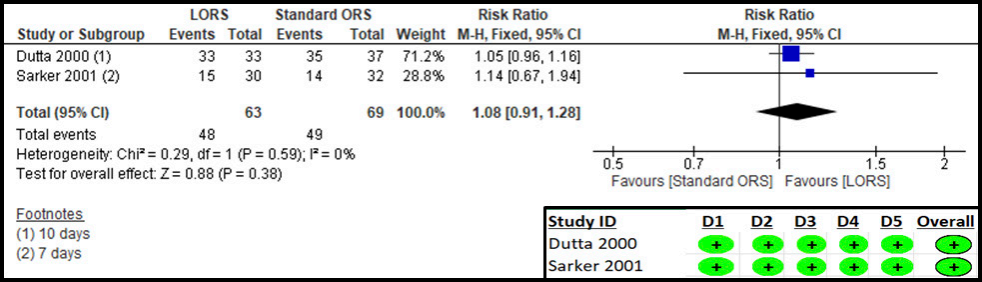
Forest plot and RoB 2 assessment for patients cured.

Two studies [23,31] reported on the outcome of the number of patients cured (defined as cessation of diarrhoea). The RoB 2 assessment suggested a low risk of bias in all domains for both studies (**Figure 8**). The results suggest that there was no significant difference between the LORs and standard ORS groups (n = 132; RR = 1.08; 95% CI = 0.91, 1.28) (**Figure 3**, **Table 3**).

One study with 70 participants reported on the mean duration of diarrhoea [23]. The RoB 2 assessment suggested a low risk of bias across all domains (Figure S14 in the **Online Supplementary Document**). The result suggests a significant reduction in the duration of diarrhoea in the LORS group compared to the standard ORS group (n = 70; MD = −30.60; 95% CI = −48.95, −12.25) (**Table 3**).

#### Secondary outcomes

One study reported on stool output (g/kg/d) [23]. The RoB 2 assessment suggested a low risk of bias (Figure S15 in the **Online Supplementary Document**). The result suggests there was a significant reduction in the LORS group, compared to the standard ORS group (n = 70; MD = −14.00; 95% CI = −26.63, −1.37) (**Table 3**).

The very same study also reported on ORS intake (ml/kg/d) for persistent diarrhoea [23], and the RoB 2 assessment suggested a low risk of bias (Figure S16 in the **Online Supplementary Document**). The result suggests there was a significant reduction in the LORS group, compared to the standard ORS group (n = 70; MD = −21.40; 95% CI = −41.01, −1.79) (**Table 3**).

## DISCUSSION

The results of our systematic review and meta-analysis indicate that LORS is either comparable or better to standard ORS for treating children under 10 years of age with acute watery or persistent diarrhoea. Overall, LORS significantly reduced the duration of diarrhoea, stool output, and ORS intake for acute diarrhoea, and significantly reduced the duration of diarrhoea, stool output, and ORS intake for persistent diarrhoea.

These findings are consistent with previous recommendations and systematic reviews [2,15,32]. The WHO last published its guidelines in 2005, identifying LORS as more efficacious compared to the standard ORS formulation, and all countries were advised to manufacture and use the LORS solution and there was only one trial that has been conducted since then [32]. Similarly, Rupani et al. [2] and Hahn et al. [15] recommended the use of LORS based on their analysis.

We assessed the overall risk of bias in our evidence using the RoB 2 tool. Dutta et al. [27], Khan et al. [29], Sarker et al. [31], and Dutta et al. [23] were at low risk of bias in all domains, which improves the reliability of their findings. Meanwhile, there were some concerns regarding missing outcome data for two other studies [25,28], potentially introducing uncertainty into their reported results. El-Mougi et al. [30] showed some concerns for randomisation process and deviations from intended interventions and a high risk of bias regarding measurement of the outcome of interest, suggesting a need for cautious interpretation of its outcomes. A WHO-led study [24] had some concerns for the randomisation process, while the study by Alam et al. [26] raised some concerns randomisation process and deviation from intended interventions. These nuanced assessments underscore the importance of critically evaluating the impact of bias on study outcomes to ensure a more comprehensive understanding of their findings.

We determined the quality of evidence using the GRADE approach (**Table 2**, **Table 3**) [21]. For acute diarrhoea, low-quality evidence indicated no substantial difference in patients cured, treatment failure, and frequency of unscheduled intravenous therapy. The evidence was downgraded due to high heterogeneity, some concerns in RoB 2, or if only one study was present in the outcome. Very low-quality evidence indicated a substantial reduction of stool output with LORS, with the evidence being downgraded due to high risk in RoB2 and high heterogeneity. Moderate-quality evidence indicated a substantial reduction in the duration of diarrhoea with LORS. Evidence was downgraded based on high-risk assessment on ROB 2 and high heterogeneity. Meanwhile, moderate-quality evidence indicated a substantial reduction in ORS intake; the evidence was downgraded based on some concerns in the RoB 2 assessment.

The introduction of ORS has been a landmark in the management of diarrhoea, significantly reducing morbidity and mortality. Literature suggests that the proper use of ORS could resolve dehydration in up to 90% of diarrhoea cases and prevent up to 93% of diarrhoea-related deaths [33,34]. Despite its proven efficacy, widespread adoption of ORS, especially in primary care settings remains a challenge. Community-based interventions, encompassing public education, health care worker training, improved distribution strategies, and the co-promotion of zinc alongside ORS, have demonstrated promising outcomes in enhancing ORS use [13,33]. Collaborative efforts will be instrumental in guaranteeing easy access to LORS sachets, thereby maximising its impact on public health and further solidifying its status as a key intervention in diarrhoea management.

Future research and initiatives should prioritise areas that enhance the coverage and use of LORS. These may include investigating novel strategies for community-level dissemination, assessing the effectiveness of different educational interventions targeting health care providers and caregivers, and evaluating the long-term impact of LORS implementation on diarrhoea morbidity and mortality rates.

In clinical settings, LORS can be considered a viable alternative to standard ORS, which can have significant beneficial effects on the management of diarrhoea resulting in reduced treatment failure rates, higher cure rates, and reduced stool outputs, thus resulting in shorter hospital stays and lower health care costs. This is particularly useful in settings with restricted access to intravenous fluid therapies and incorporating LORS into clinical practice guidelines could potentially improve treatment outcomes, reduce the burden on health care facilities, and contribute to overall cost savings.

Our review has several strengths, the first being a comprehensive search strategy and the exclusive inclusion of randomised controlled trials in our analysis. We also based our methodology on the Cochrane Handbook, according to which we performed log approximation when studies reported the outcome in geometric and arithmetic means. Lastly, we also conducted subgroup analysis based on sensitivity, reporting time points, and study region.

Some noteworthy limitations are that several studies in our review were limited to small sample sizes. There was also only one new study published since the previous Cochrane review was conducted and the WHO guidelines were developed. The scarcity of evidence, along with limited sample sizes, can compromise the robustness and generalisability of our results, which calls for exercising caution while interpreting these findings for broader populations or clinical contexts. Our inclusion of only English-language studies may have also introduced language bias, potentially leading to the omission of relevant studies published in other languages. Additionally, the heterogeneity in our analysis was high, unchanged on subgroup analysis, suggesting inherent variability among the included studies that was not adequately explained by our subgroup classifications. Our subgroup analysis based on the study region showed that all studies were conducted in LMICs, with only one study coming from a mixed setting, preventing us from generalising our findings. Lastly, we initially aimed to assess a patient population of up to 10 years of age; however, all included studies had a patient population aged five years or below. This highlight a need for further research in children beyond this age group in order to provide evidence for mitigation strategies in high burden areas.

## CONCLUSIONS

Our review summarises the data from studies conducted since the year 1990 and provides evidence that LORS leads to a significant decrease in the duration of diarrhoea, stool output, and ORS intake. Alongside other available evidence and expert discussions, our findings will contributed to the decision of the WHO in determining the efficacy of LORS compared to standard ORS. We therefore recommend that LORS should be used widely in place of the old standard formulation.

## Additional material


Online Supplementary Document
