# Supplementary material for: Low-osmolarity oral rehydration solution for childhood diarrhoea: A systematic review and meta-analysis
Source: J Glob Health. 2024 Dec 6;14:04166. doi: 10.7189/jogh.14.04166 (PMC11622343; doi:10.7189/jogh.14.04166)
Supplement: Online Supplementary Document [file jogh-14-04166-s001.pdf]

Online Supplementary Document:

SEARCH STRATEGY:

PubMed:

Table S1: PubMed Search Strategy

Diarrhea	Children up to 10 years	LORS
("Diarrhea"[MeSH Terms] OR ("Diarrhea"[Title/Abstract] OR "diarrhoea"[Title/Abstract])) OR "diarrhea, infantile"[MeSH Terms] OR "infantile diarrhea*"[Title/Abstract] OR "childhood diarrhea*"[Title/Abstract])	("child"[MeSH Terms] OR "child, preschool"[MeSH Terms] OR ("child*"[Title/Abstract] OR "preschool*"[Title/Abstract] OR "schoolchild*"[Title/Abstract] OR "school age"[Title/Abstract] OR "kid"[Title/Abstract] OR "toddler*"[Title/Abstract]) OR "infant"[MeSH Terms] OR ("infant*"[Title/Abstract] OR "Infancy"[Title/Abstract] OR "Baby"[Title/Abstract] OR "Babies"[Title/Abstract] OR "newborn*"[Title/Abstract] OR "neonat*"[Title/Abstract] OR "Preterm"[Title/Abstract] OR "prematur*"[Title/Abstract]) OR "pediatrics"[MeSH Terms] OR ("pediatric*"[Title/Abstract] OR "paediatric*"[Title/Abstract]) OR "schools, nursery"[MeSH Terms] OR ("nursery school"[Title/Abstract] OR "kindergar*"[Title/Abstract]))	(("osmolar*"[Title/Abstract] OR "Osmolar Concentration"[MeSH Terms] OR (("Low"[All Fields] AND ("Osmolar Concentration"[MeSH Terms] OR ("osmolar"[All Fields] AND "concentration"[All Fields]) OR "Osmolar Concentration"[All Fields] OR "osmolarities"[All Fields] OR "osmolarity"[All Fields] OR "osmolar"[All Fields])) AND "oral rehydration salt*"[Title/Abstract]) OR (("Low"[All Fields] AND ("Osmolar Concentration"[MeSH Terms] OR ("osmolar"[All Fields] AND "concentration"[All Fields]) OR "Osmolar Concentration"[All Fields] OR "osmolarities"[All Fields] OR "osmolarity"[All Fields] OR "osmolar"[All Fields])) AND "oral rehydration solution*"[Title/Abstract]) OR ("Low"[All Fields] AND "osmolar ors"[Title/Abstract]) OR "oral rehydration solution*"[Title/Abstract] OR "ORS"[Title/Abstract] OR "oral rehydration

		salt*"[Title/Abstract] OR "oral rehydration therap*"[Title/Abstract] OR "ORT"[Title/Abstract] OR "oral rehydration fluid"[Title/Abstract]) AND ("dehydration"[MeSH Terms] OR "dehydrat*"[Title/Abstract]))

Cochrane Central Register of Controlled Trials:

Table S2: Cochrane Search Strategy

Children up to 10 years	Diarrhea	LORS
#1 MeSH descriptor: [Child] explode all trees<br><br>#2 MeSH descriptor: [Child, Preschool] explode all trees<br><br>#3 (Child* OR preschool* OR Schoolchild* OR “school age” OR Kid OR Kids OR toddler*):ti,ab,kw<br><br>#4 MeSH descriptor: [Infant] explode all trees<br><br>#5 (Infant* OR Infancy OR Baby OR Babies OR Newborn* OR Neonat* OR Preterm OR Prematur*):ti,ab,kw<br><br>#6 MeSH descriptor: [Pediatrics] explode all trees<br><br>#7 (Pediatric* OR Paediatric*):ti,ab,kw<br><br>#8 MeSH descriptor: [Schools, Nursery] explode all trees	#11 MeSH descriptor: [Diarrhea] explode all trees<br><br>#12 ((Diarrhea OR diarrhoea)):ti,ab,kw<br><br>#13 MeSH descriptor: [Diarrhea, Infantile] explode all trees<br><br>#14 (“Infantile Diarrhea*”):ti,ab,kw<br><br>#15 (“Childhood Diarrhea*”):ti,ab,kw<br><br>#16: #11 OR #12 OR #13 OR #14 OR #15<br><br>#17: #10 AND #16	#18 (Osmolar*):ti,ab,kw<br><br>#19 MeSH descriptor: [Osmolar Concentration] explode all trees<br><br>#20 (Low osmolar oral rehydration salt*):ti,ab,kw 3<br><br>#21 (Low osmolar oral rehydration solution*):ti,ab,kw<br><br>#22 (Low osmolar ORS):ti,ab,kw<br><br>#23 (Oral rehydration solution*):ti,ab,kw<br><br>#24 (ORS):ti,ab,kw<br><br>#25 (Oral rehydration salt*):ti,ab,kw<br><br>#26 (Oral rehydration therap*):ti,ab,kw<br><br>#27 (ORT):ti,ab,kw

#9 (Nursery school OR Kindergar*):ti,ab,kw<br><br>#10: #1 OR #2 OR #3 OR #4 OR #5 OR #6 OR #7 OR #8 OR #9		#28 (Oral rehydration fluid):ti,ab,kw<br><br>#29: #18 OR #19 OR #20 OR #21 OR #22 OR #23 OR #24 OR #25 OR #26 OR #27 OR #28<br><br>#30 MeSH descriptor: [Dehydration] explode all trees<br><br>#31 (Dehydrat*):ti,ab,kw<br><br>#32: #30 OR #31<br><br>#33: #29 AND #32<br><br>#34: #17 AND #33

Scopus:

Table S3: Scopus Search Strategy

Diarrhea	Children up to 10 years	LORS
TITLE-ABS-KEY ((diarrhea OR dehydrat*))	(TITLE-ABS-KEY ((child* OR "preschool child*" OR schoolchild* OR kid OR kids OR toddler* OR infant OR infants OR "Infancy" OR "Baby" OR "Babies" OR "newborn*" OR "neonat*" OR preterm OR prematur* OR "pediatric*" OR "paediatric*" OR kinderg*))	TITLE-ABS-KEY ((osmolar* OR "Osmolar Concentration" OR "Low osmolar oral rehydration salt*" OR "Low osmolar oral rehydration solution*" OR "Low osmolar ORS" OR "Oral rehydration solution*" OR ors OR "Oral rehydration salt*" OR "Oral rehydration therap*" OR ort OR "Oral rehydration fluid")))

CINAHL:

Table S4: CINAHIL Search Strategy

Diarrhea	Children up to 10 years	LORS
(((MH "Diarrhea") OR MH dehydration OR MH Dysentery OR MH Giardiasis OR "blood in stool" OR Dysenter* OR “Abdominal distension ” OR “loose motion”))	((Infant* OR toddler* OR baby OR babies OR preschool OR newborn OR neonate* OR kindergarten OR under-10 OR under-ten OR “under ten” OR kid OR kids OR paediatr* OR pediatr*))	((Osmolar* OR "Osmolar Concentration" OR “Low osmolar oral rehydration salt*” OR “Low osmolar oral rehydration solution*” OR “Low osmolar ORS” OR “Oral rehydration solution*” OR ORS OR “Oral rehydration salt*” OR “Oral rehydration therap*” OR ORT OR “Oral rehydration fluid”))

Clinicaltrials.gov

Table S5: Clinicaltrials.gov Search Strategy

Diarrhea	Children up to 10 years	LORS
"Diarrhea" OR "Acute" OR "Persistent" AND "Diarrhea" OR "diarrhoea" OR "diarrhea, infantile" OR "infantile diarrhea*" OR "childhood diarrhea*"	"child" OR "child, preschool" OR "child*" OR "preschool*" OR "schoolchild*" OR "school age" OR "Kid" OR "Kids" OR "toddler*" OR "infant" OR "infant*" OR "Infancy" OR "Baby" OR "Babies" OR "newborn*" OR "neonat*" OR "Preterm" OR "prematur*" OR "pediatrics" OR "pediatric*" OR "paediatric*" OR "schools, nursery" OR "nursery school" OR "kindergar*"	"osmolar*" OR "Osmolar Concentration" OR "Low" AND "Osmolar Concentration" OR "osmolar" AND "concentration" OR "Osmolar Concentration" OR "osmolarities" OR "osmolarity" OR "osmolar" AND "oral rehydration salt*" OR "Low" AND "Osmolar Concentration" OR "osmolar" AND

		"concentration" OR "Osmolar Concentration" OR "osmolarities" OR "osmolarity" OR "osmolar" AND "oral rehydration solution*" OR "Low" AND "osmolar ORS" OR "oral rehydration solution*" OR "ORS" OR "oral rehydration salt*" OR "oral rehydration therap*" OR "ORT" OR "oral rehydration fluid" AND "Dehydration" OR "dehydrat*"

(Figures and Tables)

Table S6: Actual values for duration of diarrhea

	**Mean (SD)**	
**Study ID**	**Intervention**	**Control**
Alam 2000	27.3(1.71) **	31.1(1.89) **
Choice 2001	56(36.9)	54(36.5)
Dutta 2001	41.5(25.1)	66.4(32.3)
El-Mougi 1994	31(19)	48(33)
Santosham 1996	41(30)	44(33)
WHO 1995	33(30-39) *	41(37-46) *

*Geometric mean (95% CI)
**Geometric mean (SD)

Table S7: Actual values for mean stool output

	**Mean (SD)**	
**Study ID**	**Intervention**	**Control**
Choice 2001	320(331.92)	331(329)
Dutta 2001	141.2(28.6)	164.2(44.7)
El-Mougi 1994	165(52)	260(114)
Khan 2005	159(162.6) **	156(143) **
Santosham 1996	80(67-95) *	98(83-117)
WHO 1995	107(90-127) *	149(128-173) *

*Geometric mean (95% CI)
** Originally reported median (IQR)

Table S8: Actual values for mean ORS Intake

	**Mean (SD)**	
**Study ID**	**Intervention**	**Control**
Choice 2001	424(369.3)	443(365.5)
Dutta 2001	301.05(51.22)	287.1(54.8)
El-Mougi 1994	219(70)	262(110)
Khan 2005	220.6(210.2) **	220(138.4) **
Santosham 1996	116(87-155) *	160(119-214) *
WHO 1995	210(191-230) *	246(225-269) *

*Geometric mean (95% CI)
** Originally reported median (IQR)

Table S9: Estimates based on sensitivity analysis.

**Outcome**	**No. of studies**	**Estimate**
3.1 Log-approximated duration of diarrhea	4	MD -0.30 (95% CI: -0.44 to -0.15)
4.1 Log-approximated stool output	5	MD -0.23 (95% CI: -0.32 to -0.13)
5.1 Frequency of unscheduled IV fluids	2	RR 1.14 (95% CI: 0.31 to 4.21)
6.1 Log-approximated ORS intake	4	MD -0.16 (95% CI: -0.39 to 0.06)

Figure S1: Forest Plot and ROB 2 Assessment for Treatment Failure:

Study or Subgroup	LORS Events	LORS Total	Standard ORS Events	Standard ORS Total	Weight	Risk Ratio M-H, Fixed, 95% CI
Santosham 1996	1	94	8	96	100.0%	0.13 [0.02, 1.00]
**Total (95% CI)**		**94**		**96**	**100.0%**	**0.13 [0.02, 1.00]**
Total events	1		8			

Heterogeneity: Not applicable
Test for overall effect: Z = 1.96 (P = 0.05)

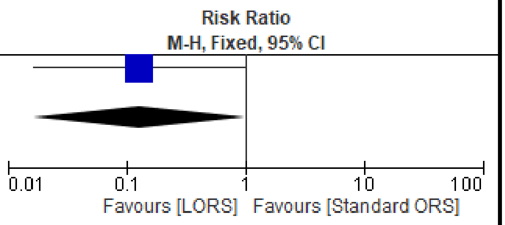

Study ID	D1	D2	D3	D4	D5	Overall
Santosham 1996	+	+	!	+	+	!

Forest plots for Duration of Diarrhea Sub-groups:

Figure S2: Log-approximated Duration of Diarrhea – Sensitivity:

Study or Subgroup	LORS Mean	LORS SD	LORS Total	Standard ORS Mean	Standard ORS SD	Standard ORS Total	Weight	Mean Difference IV, Random, 95% CI
Choice 2001	3.8	0.6	341	3.9	0.6	334	28.5%	-0.10 [-0.19, -0.01]
Dutta 2001	3.5	0.5	32	4.1	0.4	32	18.4%	-0.60 [-0.82, -0.38]
Santosham 1996	3.4	0.6	82	3.7	0.6	78	21.1%	-0.30 [-0.49, -0.11]
WHO 1995	3.4	0.06	221	3.7	0.05	218	31.9%	-0.30 [-0.31, -0.29]
Total (95% CI)			676			662	100.0%	-0.30 [-0.44, -0.15]

Heterogeneity: Tau² = 0.02; Chi² = 25.64, df = 3 (P < 0.0001); I² = 88%
Test for overall effect: Z = 3.99 (P < 0.0001)

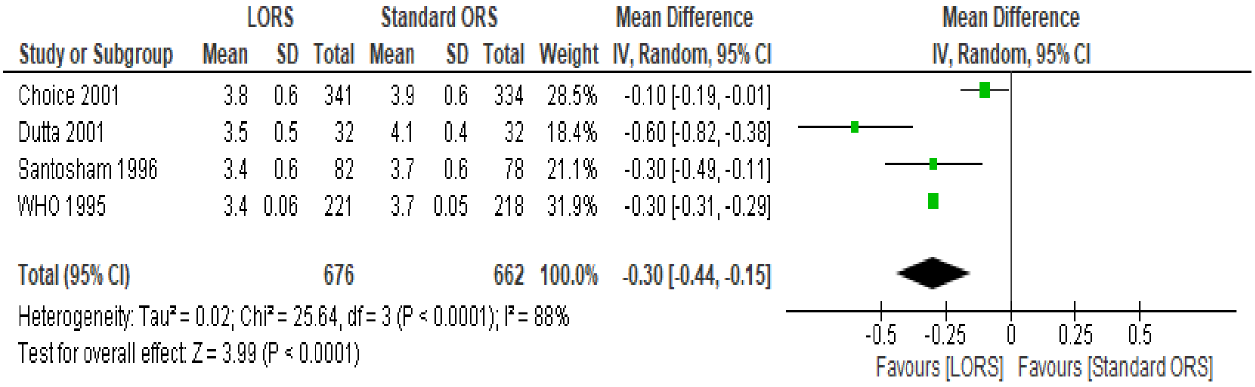

Figure S3: Log-approximated Duration of Diarrhea - Subgroup based on reporting time:

Study or Subgroup	LORS Mean	LORS SD	LORS Total	Standard ORS Mean	Standard ORS SD	Standard ORS Total	Weight	Mean Difference IV, Random, 95% CI
**1.3.1 Total**								
Alam 2000	3.3	0.5	88	3.4	0.6	82	16.8%	-0.10 [-0.27, 0.07]
Choice 2001	3.8	0.6	341	3.9	0.6	334	21.1%	-0.10 [-0.19, -0.01]
**Subtotal (95% CI)**			**429**			**416**	**38.0%**	**-0.10 [-0.18, -0.02]**
Heterogeneity: Tau² = 0.00; Chi² = 0.00, df = 1 (P = 1.00); I² = 0%								
Test for overall effect: Z = 2.46 (P = 0.01)								
**1.3.2 After Initiation of therapy**								
Dutta 2001	3.5	0.5	32	4.1	0.4	32	13.8%	-0.60 [-0.82, -0.38]
El- Mougi 1994	3.2	0.5	20	3.8	0.6	21	8.9%	-0.60 [-0.94, -0.26]
Santosham 1996	3.4	0.6	82	3.7	0.6	78	15.7%	-0.30 [-0.49, -0.11]
WHO 1995	3.4	0.06	221	3.7	0.05	218	23.6%	-0.30 [-0.31, -0.29]
**Subtotal (95% CI)**			**355**			**349**	**62.0%**	**-0.41 [-0.56, -0.25]**
Heterogeneity: Tau² = 0.02; Chi² = 10.03, df = 3 (P = 0.02); I² = 70%								
Test for overall effect: Z = 5.14 (P < 0.00001)								
**Total (95% CI)**			**784**			**765**	**100.0%**	**-0.29 [-0.42, -0.16]**

Heterogeneity: Tau² = 0.02; Chi² = 34.13, df = 5 (P < 0.00001); I² = 85%
Test for overall effect: Z = 4.50 (P < 0.00001)
Test for subgroup differences: Chi² = 11.91, df = 1 (P = 0.0006), I² = 91.6%

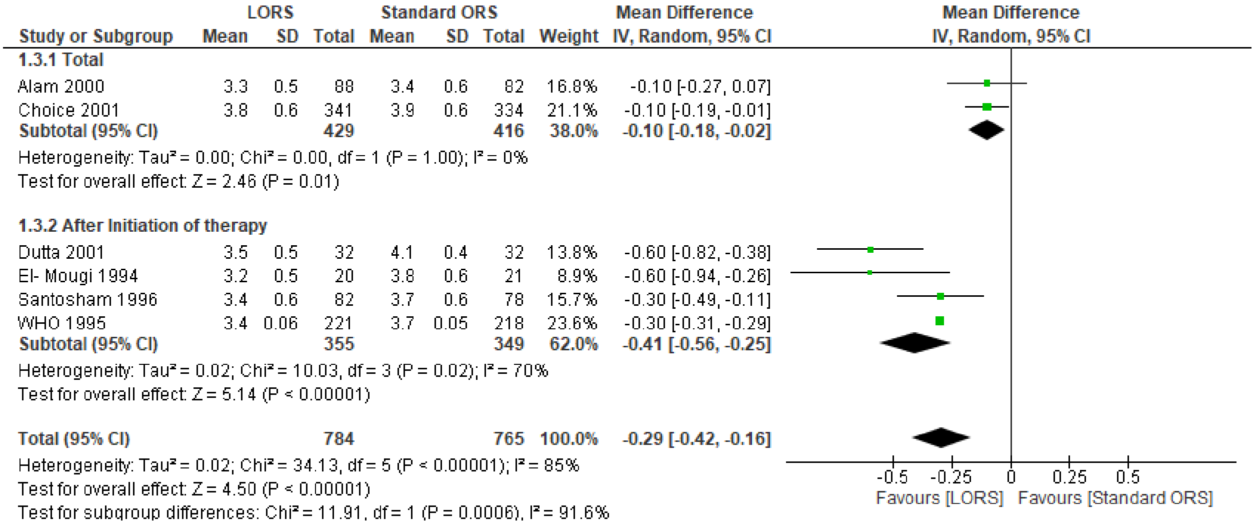

Figure S4: Log-approximated Duration of Diarrhea – Subgroup based on study region:

Study or Subgroup	LORS Mean	LORS SD	LORS Total	Standard ORS Mean	Standard ORS SD	Standard ORS Total	Weight	Mean Difference IV, Random, 95% CI
**1.3.1 LMIC**								
Alam 2000 (1)	3.3	0.5	88	3.4	0.6	82	16.8%	-0.10 [-0.27, 0.07]
Choice 2001 (2)	3.8	0.6	341	3.9	0.6	334	21.1%	-0.10 [-0.19, -0.01]
Dutta 2001 (3)	3.5	0.5	32	4.1	0.4	32	13.8%	-0.60 [-0.82, -0.38]
El- Mougi 1994 (4)	3.2	0.5	20	3.8	0.6	21	8.9%	-0.60 [-0.94, -0.26]
Santosham 1996 (5)	3.4	0.6	82	3.7	0.6	78	15.7%	-0.30 [-0.49, -0.11]
**Subtotal (95% CI)**			**563**			**547**	**76.4%**	**-0.31 [-0.51, -0.11]**
Heterogeneity: Tau² = 0.04; Chi² = 25.04, df = 4 (P < 0.0001); I² = 84%								
Test for overall effect: Z = 3.07 (P = 0.002)								
**1.3.2 Mixed (LMIC + HIC)**								
WHO 1995 (6)	3.4	0.06	221	3.7	0.05	218	23.6%	-0.30 [-0.31, -0.29]
**Subtotal (95% CI)**			**221**			**218**	**23.6%**	**-0.30 [-0.31, -0.29]**
Heterogeneity: Not applicable								
Test for overall effect: Z = 56.94 (P < 0.00001)								
**Total (95% CI)**			**784**			**765**	**100.0%**	**-0.29 [-0.42, -0.16]**
Heterogeneity: Tau² = 0.02; Chi² = 34.13, df = 5 (P < 0.00001); I² = 85%								
Test for overall effect: Z = 4.50 (P < 0.00001)								
Test for subgroup differences: Chi² = 0.01, df = 1 (P = 0.92), I² = 0%								

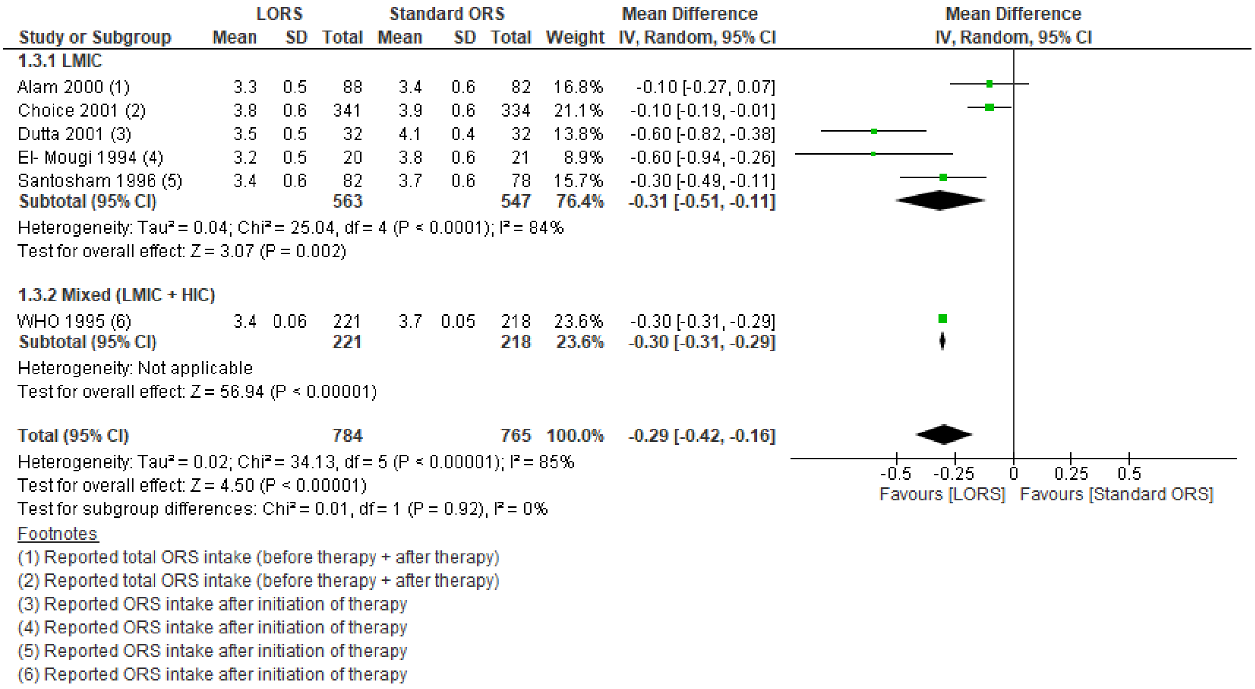

Footnotes
(1) Reported total ORS intake (before therapy + after therapy)
(2) Reported total ORS intake (before therapy + after therapy)
(3) Reported ORS intake after initiation of therapy
(4) Reported ORS intake after initiation of therapy
(5) Reported ORS intake after initiation of therapy
(6) Reported ORS intake after initiation of therapy

Forest Plots for Stool Output Subgroups:

Figure S5: Log-approximated Stool Output Sensitivity:

Study or Subgroup	LORS Mean	LORS SD	LORS Total	Standard ORS Mean	Standard ORS SD	Standard ORS Total	Weight	Mean Difference IV, Random, 95% CI
Choice 2001	5.7	0.5	341	5.8	0.8	334	20.4%	-0.10 [-0.20, 0.00]
Dutta 2001 (1)	4.9	0.2	32	5.1	0.3	32	18.2%	-0.20 [-0.32, -0.08]
Khan 2005 (2)	4.7	0.8	73	5.04	0.78	71	8.9%	-0.34 [-0.60, -0.08]
Santosham 1996	4.3	0.08	86	4.5	0.08	83	26.1%	-0.20 [-0.22, -0.18]
WHO 1995 (3)	4.67	0.08	221	5	0.07	218	26.4%	-0.33 [-0.34, -0.32]
**Total (95% CI)**			**753**			**738**	**100.0%**	**-0.23 [-0.32, -0.13]**
Heterogeneity: Tau² = 0.01; Chi² = 100.00, df = 4 (P < 0.00001); I² = 96%								
Test for overall effect: Z = 4.69 (P < 0.00001)								

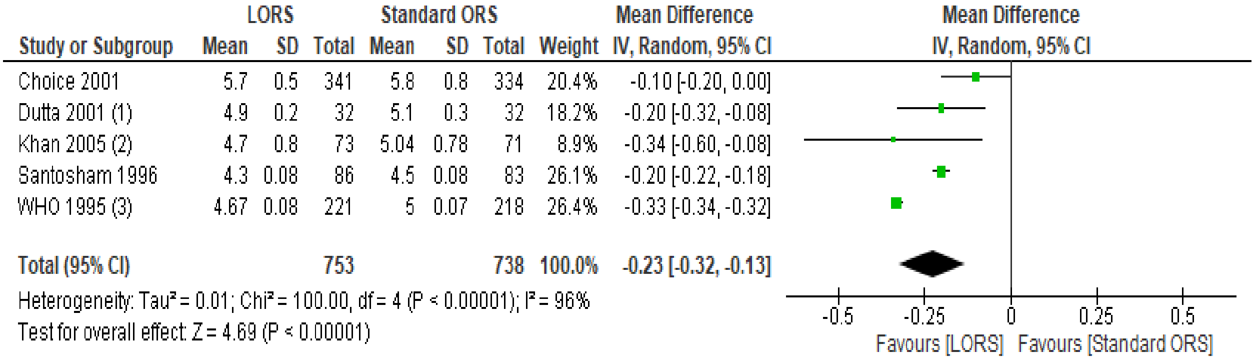

Footnotes
(1) Reported at recovery
(2) Reported as total
(3) Reported as total

Figure S6: Log-approximated Stool Output – Subgroup based on reporting time:

Study or Subgroup	LORS Mean	LORS SD	LORS Total	Standard ORS Mean	Standard ORS SD	Standard ORS Total	Weight	Mean Difference IV, Random, 95% CI
**1.1.1 Rehydration phase**								
Santosham 1996	2.39	0.14	94	2.7	0.131	96	100.0%	-0.31 [-0.35, -0.27]
**Subtotal (95% CI)**			**94**			**96**	**100.0%**	**-0.31 [-0.35, -0.27]**
Heterogeneity: Not applicable								
Test for overall effect: Z = 15.75 (P < 0.00001)								
**1.1.3 at 24 hrs**								
Choice 2001	4.7	0.38	341	4.8	0.65	334	19.8%	-0.10 [-0.18, -0.02]
Dutta 2001	4.2	0.3	32	4.6	0.4	32	10.0%	-0.40 [-0.57, -0.23]
El- Mougi 1994	5.05	0.3	20	5.56	0.41	21	7.3%	-0.51 [-0.73, -0.29]
Khan 2005	3.83	0.6	73	3.9	0.5	71	9.6%	-0.07 [-0.25, 0.11]
Santosham 1996	4.3	0.08	86	4.5	0.08	83	26.3%	-0.20 [-0.22, -0.18]
WHO 1995	4.17	0.05	221	4.45	0.05	218	27.0%	-0.28 [-0.29, -0.27]
**Subtotal (95% CI)**			**773**			**759**	**100.0%**	**-0.23 [-0.30, -0.16]**
Heterogeneity: Tau² = 0.00; Chi² = 65.10, df = 5 (P < 0.00001); I² = 92%								
Test for overall effect: Z = 6.57 (P < 0.00001)								
**1.1.4 At 72 h**								
Dutta 2001	3.17	0.58	32	4.5	0.66	32	100.0%	-1.33 [-1.63, -1.03]
**Subtotal (95% CI)**			**32**			**32**	**100.0%**	**-1.33 [-1.63, -1.03]**
Heterogeneity: Not applicable								
Test for overall effect: Z = 8.56 (P < 0.00001)								
**1.1.5 Total**								
Choice 2001	5.7	0.5	341	5.8	0.8	334	27.4%	-0.10 [-0.20, 0.00]
Dutta 2001	4.9	0.2	32	5.1	0.3	32	25.4%	-0.20 [-0.32, -0.08]
Khan 2005	4.7	0.8	73	5.04	0.78	71	15.1%	-0.34 [-0.60, -0.08]
WHO 1995	4.67	0.08	221	5	0.07	218	32.0%	-0.33 [-0.34, -0.32]
**Subtotal (95% CI)**			**667**			**655**	**100.0%**	**-0.24 [-0.37, -0.10]**
Heterogeneity: Tau² = 0.02; Chi² = 23.44, df = 3 (P < 0.0001); I² = 87%								
Test for overall effect: Z = 3.35 (P = 0.0008)								

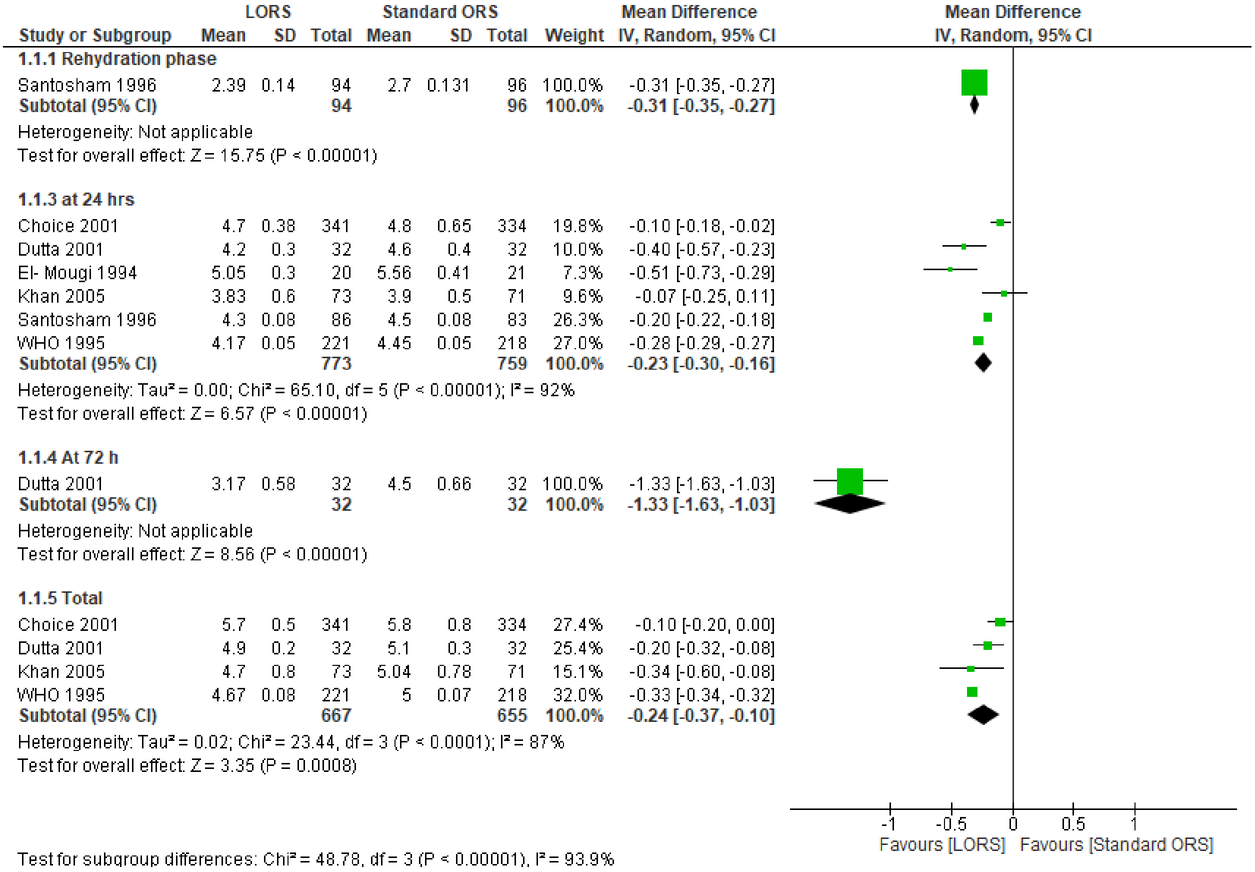

Test for subgroup differences: Chi² = 48.78, df = 3 (P < 0.00001), I² = 93.9%

Figure S7: Log-approximated Stool Output - Subgroup based on study region:

Study or Subgroup	LORS Mean	LORS SD	LORS Total	Standard ORS Mean	Standard ORS SD	Standard ORS Total	Weight	Mean Difference IV, Random, 95% CI
**1.1.1 LMIC**								
Choice 2001 (1)	5.7	0.5	341	5.8	0.8	334	18.4%	-0.10 [-0.20, 0.00]
Dutta 2001 (2)	4.9	0.2	32	5.1	0.3	32	16.4%	-0.20 [-0.32, -0.08]
El- Mougi 1994 (3)	5.05	0.3	20	5.56	0.41	21	9.9%	-0.51 [-0.73, -0.29]
Khan 2005 (4)	4.7	0.8	73	5.04	0.78	71	8.1%	-0.34 [-0.60, -0.08]
Santosham 1996 (5)	4.3	0.08	86	4.5	0.08	83	23.5%	-0.20 [-0.22, -0.18]
**Subtotal (95% CI)**			**552**			**541**	**76.3%**	**-0.22 [-0.31, -0.13]**
Heterogeneity: Tau² = 0.01; Chi² = 12.59, df = 4 (P = 0.01); I² = 68%								
Test for overall effect: Z = 4.86 (P < 0.00001)								
**1.1.2 Mixed (LMIC + HIC)**								
WHO 1995 (6)	4.67	0.08	221	5	0.07	218	23.7%	-0.33 [-0.34, -0.32]
**Subtotal (95% CI)**			**221**			**218**	**23.7%**	**-0.33 [-0.34, -0.32]**
Heterogeneity: Not applicable								
Test for overall effect: Z = 46.01 (P < 0.00001)								
**Total (95% CI)**			**773**			**759**	**100.0%**	**-0.25 [-0.35, -0.16]**
Heterogeneity: Tau² = 0.01; Chi² = 103.74, df = 5 (P < 0.00001); I² = 95%								
Test for overall effect: Z = 5.52 (P < 0.00001)								
Test for subgroup differences: Chi² = 5.18, df = 1 (P = 0.02), I² = 80.7%								

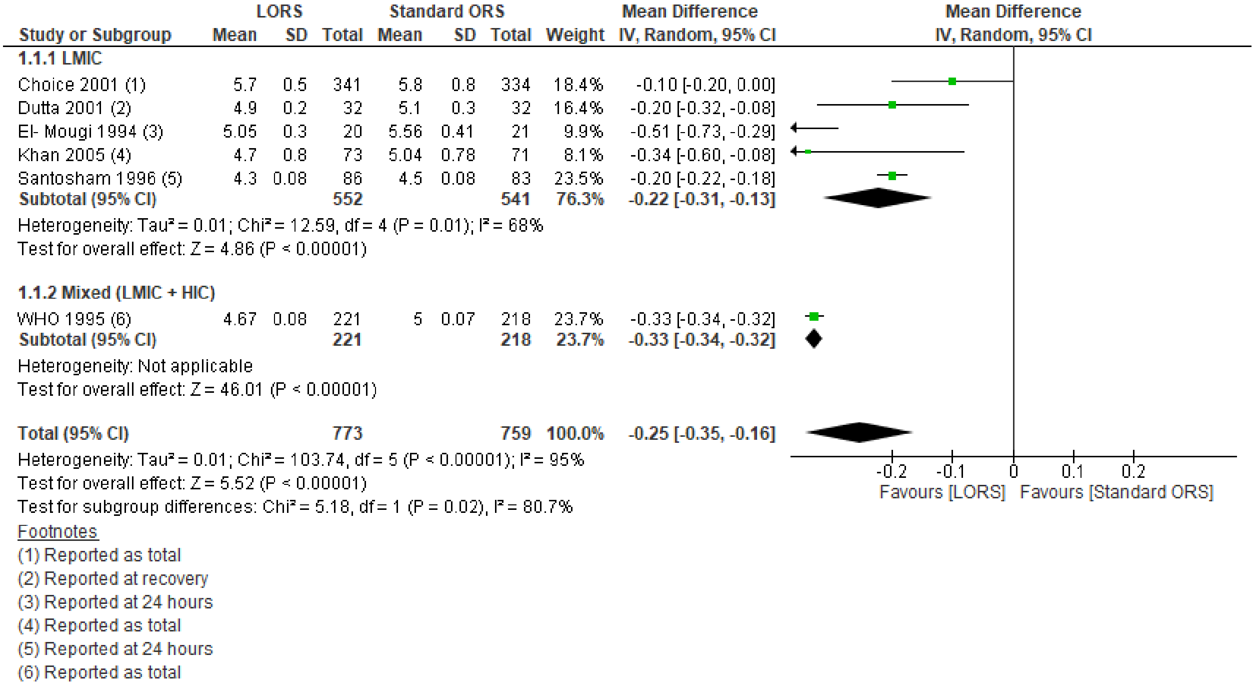

Footnotes
(1) Reported as total
(2) Reported at recovery
(3) Reported at 24 hours
(4) Reported as total
(5) Reported at 24 hours
(6) Reported as total

Forest plots for frequency of IV fluids subgroups:

Figure S8: Frequency of IVF – Sensitivity:

Study or Subgroup	LORS Events	LORS Total	Standard ORS Events	Standard ORS Total	Weight	Risk Ratio M-H, Random, 95% CI
Choice 2001	34	341	50	334	60.4%	0.67 [0.44, 1.00]
Khan 2005	8	73	3	71	39.6%	2.59 [0.72, 9.38]
**Total (95% CI)**		**414**		**405**	**100.0%**	**1.14 [0.31, 4.21]**
Total events	42		53			
Heterogeneity: Tau² = 0.69; Chi² = 3.92, df = 1 (P = 0.05); I² = 74%						
Test for overall effect: Z = 0.20 (P = 0.84)						

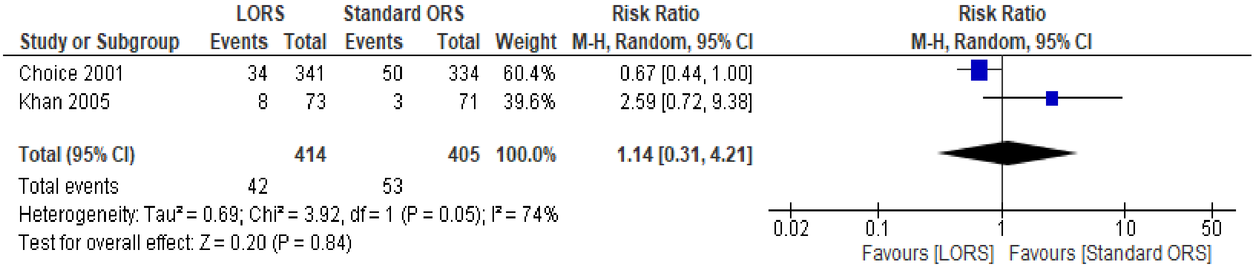

Figure S9: Frequency of IVF - Subgroup based on reporting time:

Study or Subgroup	LORS Events	LORS Total	Standard ORS Events	Standard ORS Total	Weight	Risk Ratio M-H, Fixed, 95% CI
**1.10.1 Total**						
Alam 2000	33	221	43	218	44.7%	0.76 [0.50, 1.14]
Khan 2005	8	73	3	71	3.1%	2.59 [0.72, 9.38]
**Subtotal (95% CI)**		**294**		**289**	**47.8%**	**0.88 [0.60, 1.29]**
Total events	41		46			
Heterogeneity: Chi² = 3.22, df = 1 (P = 0.07); I² = 69%						
Test for overall effect: Z = 0.66 (P = 0.51)						
**1.10.2 At 24 Hours**						
Choice 2001	34	341	50	334	52.2%	0.67 [0.44, 1.00]
**Subtotal (95% CI)**		**341**		**334**	**52.2%**	**0.67 [0.44, 1.00]**
Total events	34		50			
Heterogeneity: Not applicable						
Test for overall effect: Z = 1.95 (P = 0.05)						
**Total (95% CI)**		**635**		**623**	**100.0%**	**0.77 [0.58, 1.02]**
Total events	75		96			
Heterogeneity: Chi² = 3.91, df = 2 (P = 0.14); I² = 49%						
Test for overall effect: Z = 1.85 (P = 0.06)						
Test for subgroup differences: Chi² = 0.92, df = 1 (P = 0.34), I² = 0%						

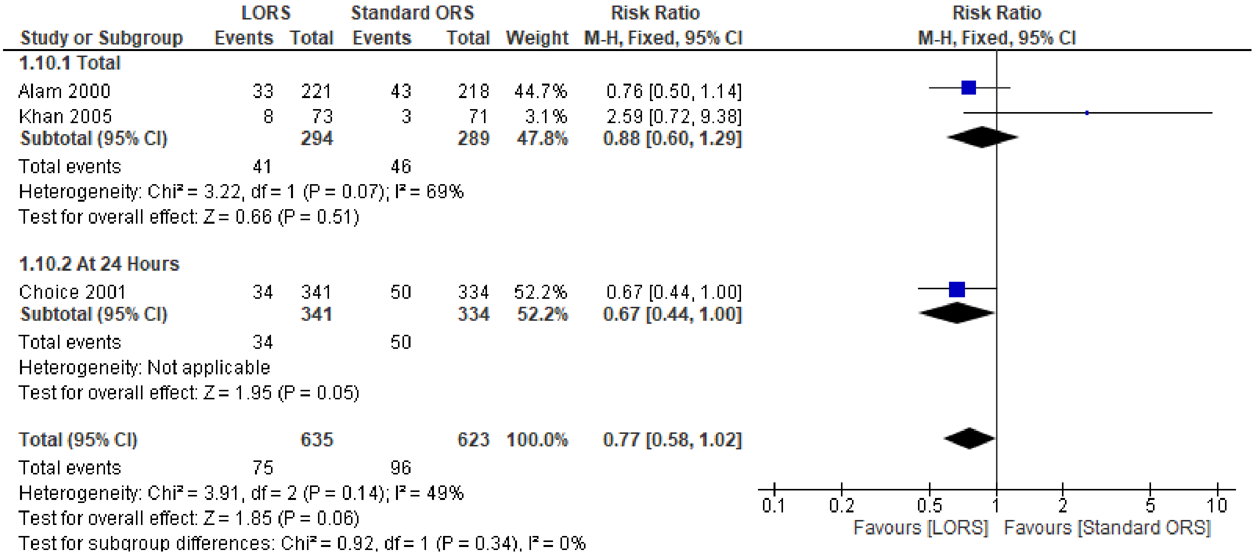

Forest plots for ORS Intake:

Figure S10: Log-approximated ORS Intake – Sensitivity:

Study or Subgroup	LORS Mean	LORS SD	LORS Total	Standard ORS Mean	Standard ORS SD	Standard ORS Total	Weight	Mean Difference IV, Random, 95% CI
Choice 2001	6.04	0.79	341	6.09	0.72	334	25.5%	-0.05 [-0.16, 0.06]
Dutta 2001	5.7	0.2	32	5.7	0.2	32	25.9%	0.00 [-0.10, 0.10]
Khan 2005	5.07	0.8	73	5.3	0.5	71	21.7%	-0.23 [-0.45, -0.01]
Santosham 1996	4.7	0.14	82	5.07	0.15	78	26.9%	-0.37 [-0.42, -0.32]
**Total (95% CI)**			**528**			**515**	**100.0%**	**-0.16 [-0.39, 0.06]**

Heterogeneity: Tau² = 0.05; Chi² = 62.49, df = 3 (P < 0.00001); I² = 95%
Test for overall effect: Z = 1.42 (P = 0.16)

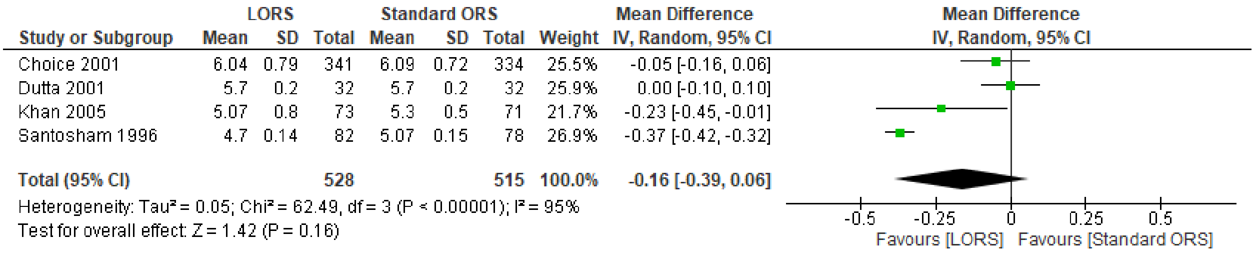

Figure S11: Log-approximated ORS Intake - Sub-group based on reporting time:

Study or Subgroup	LORS Mean	LORS SD	LORS Total	Standard ORS Mean	Standard ORS SD	Standard ORS Total	Weight	Mean Difference IV, Random, 95% CI
**1.2.1 Rehydration phase**								
Santosham 1996	4.4	0.03	94	4.4	0.04	96	100.0%	0.00 [-0.01, 0.01]
**Subtotal (95% CI)**			**94**			**96**	**100.0%**	**0.00 [-0.01, 0.01]**
Heterogeneity: Not applicable								
Test for overall effect: Z = 0.00 (P = 1.00)								
**1.2.2 Maintenance phase**								
Santosham 1996	4.7	0.14	82	5.07	0.15	78	100.0%	-0.37 [-0.42, -0.32]
**Subtotal (95% CI)**			**82**			**78**	**100.0%**	**-0.37 [-0.42, -0.32]**
Heterogeneity: Not applicable								
Test for overall effect: Z = 16.11 (P < 0.00001)								
**1.2.3 at 24 hrs**								
Choice 2001	5.2	0.4	341	5.3	0.47	334	23.0%	-0.10 [-0.17, -0.03]
Dutta 2001	4.65	0.28	32	5.21	0.2	32	19.4%	-0.56 [-0.68, -0.44]
El- Mougi 1994	5.3	0.3	20	5.5	0.4	21	13.0%	-0.20 [-0.42, 0.02]
Khan 2005	4.3	0.4	73	4.5	0.3	71	19.7%	-0.20 [-0.32, -0.08]
WHO 1995	4.9	0.02	221	5.1	0.02	218	24.9%	-0.20 [-0.20, -0.20]
**Subtotal (95% CI)**			**687**			**676**	**100.0%**	**-0.25 [-0.36, -0.13]**
Heterogeneity: Tau² = 0.01; Chi² = 43.87, df = 4 (P < 0.00001); I² = 91%								
Test for overall effect: Z = 4.32 (P < 0.0001)								
**1.2.4 Total**								
Choice 2001	6.04	0.79	341	6.09	0.72	334	25.2%	-0.05 [-0.16, 0.06]
Dutta 2001	5.7	0.2	32	5.7	0.2	32	26.7%	0.00 [-0.10, 0.10]
Khan 2005	5.07	0.8	73	5.3	0.5	71	16.2%	-0.23 [-0.45, -0.01]
WHO 1995	5.3	0.04	221	5.5	0.04	218	31.9%	-0.20 [-0.21, -0.19]
**Subtotal (95% CI)**			**667**			**655**	**100.0%**	**-0.11 [-0.24, 0.01]**
Heterogeneity: Tau² = 0.01; Chi² = 22.51, df = 3 (P < 0.0001); I² = 87%								
Test for overall effect: Z = 1.79 (P = 0.07)								

Test for subgroup differences: Chi² = 265.42, df = 3 (P < 0.00001), I² = 98.9%

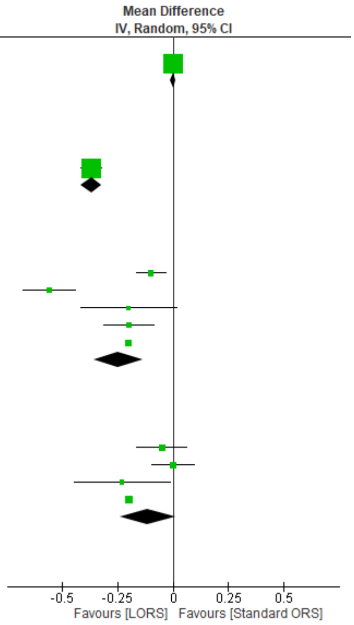

Figure S12: Log-approximated ORS Intake - Sub-group based on study region:

Study or Subgroup	LORS Mean	LORS SD	LORS Total	Standard ORS Mean	Standard ORS SD	Standard ORS Total	Weight	Mean Difference IV, Random, 95% CI
**1.2.1 LMIC**								
Choice 2001 (1)	6.04	0.79	341	6.09	0.72	334	17.2%	-0.05 [-0.16, 0.06]
Dutta 2001 (2)	5.7	0.2	32	5.7	0.2	32	18.1%	0.00 [-0.10, 0.10]
El- Mougi 1994 (3)	5.3	0.3	20	5.5	0.4	21	11.3%	-0.20 [-0.42, 0.02]
Khan 2005 (4)	5.07	0.8	73	5.3	0.5	71	11.2%	-0.23 [-0.45, -0.01]
Santosham 1996 (5)	4.7	0.14	82	5.07	0.15	78	20.7%	-0.37 [-0.42, -0.32]
**Subtotal (95% CI)**			**548**			**536**	**78.5%**	**-0.17 [-0.36, 0.03]**
Heterogeneity: Tau² = 0.04; Chi² = 62.94, df = 4 (P < 0.00001); I² = 94%								
Test for overall effect: Z = 1.71 (P = 0.09)								
**1.2.2 Mixed (LMIC + HIC)**								
WHO 1995 (6)	5.3	0.04	221	5.5	0.04	218	21.5%	-0.20 [-0.21, -0.19]
**Subtotal (95% CI)**			**221**			**218**	**21.5%**	**-0.20 [-0.21, -0.19]**
Heterogeneity: Not applicable								
Test for overall effect: Z = 52.38 (P < 0.00001)								
**Total (95% CI)**			**769**			**754**	**100.0%**	**-0.18 [-0.28, -0.07]**
Heterogeneity: Tau² = 0.01; Chi² = 76.95, df = 5 (P < 0.00001); I² = 94%								
Test for overall effect: Z = 3.28 (P = 0.001)								
Test for subgroup differences: Chi² = 0.10, df = 1 (P = 0.75), I² = 0%								

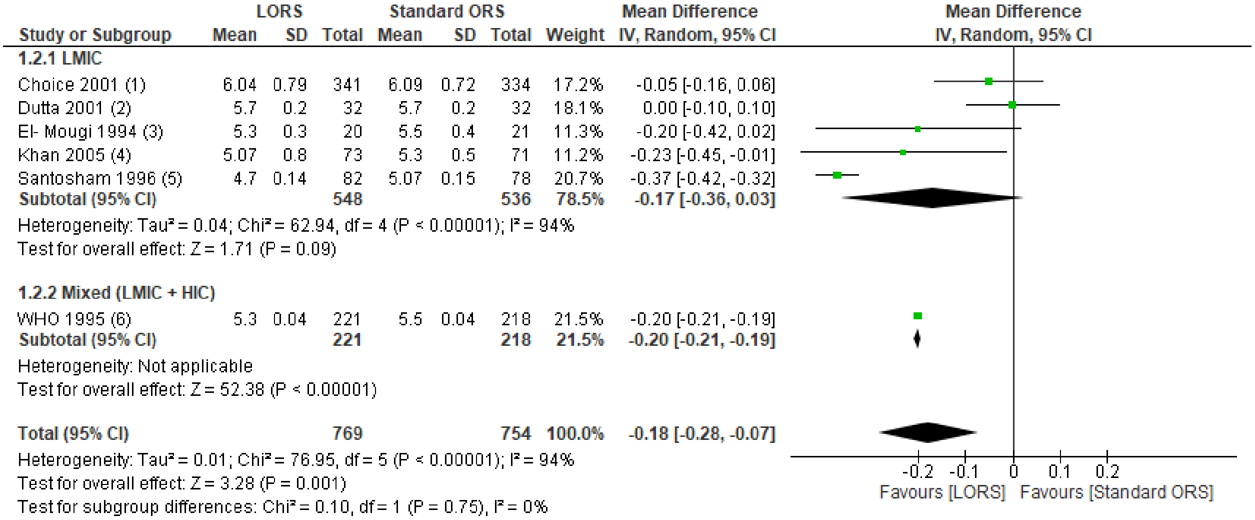

Footnotes
(1) Reported as total
(2) Reported at recovery
(3) Reported at 24 hours
(4) Reported as total
(5) Reported at Maintenance phase
(6) Reported as total

Forest Plots for Persistent Diarrhea

Figure S13: Forest Plot and ROB 2 Assessment for Patients Cured

Study or Subgroup	LORS Events	LORS Total	Standard ORS Events	Standard ORS Total	Weight	Risk Ratio M-H, Fixed, 95% CI
Dutta 2000 (1)	33	33	35	37	71.2%	1.05 [0.96, 1.16]
Sarker 2001 (2)	15	30	14	32	28.8%	1.14 [0.67, 1.94]
**Total (95% CI)**		**63**		**69**	**100.0%**	**1.08 [0.91, 1.28]**
Total events	48		49			
Heterogeneity: Chi² = 0.29, df = 1 (P = 0.59); I² = 0%						
Test for overall effect: Z = 0.88 (P = 0.38)						

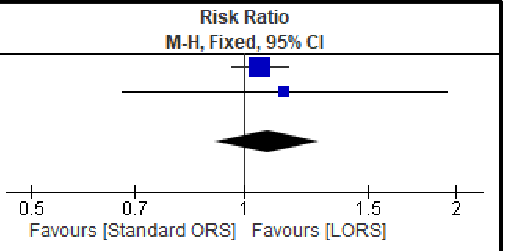

Footnotes
(1) 10 days
(2) 7 days

Study ID	D1	D2	D3	D4	D5	Overall
Dutta 2000	+	+	+	+	+	+
Sarker 2001	+	+	+	+	+	+

Figure S14: Forest Plot and ROB 2 for Duration of Diarrhea (h)

Study or Subgroup	LORS Mean	LORS SD	LORS Total	Standard ORS Mean	Standard ORS SD	Standard ORS Total	Weight	Mean Difference IV, Fixed, 95% CI
Dutta 2000	114.8	38.3	33	145.4	40	37	100.0%	-30.60 [-48.95, -12.25]
**Total (95% CI)**			**33**			**37**	**100.0%**	**-30.60 [-48.95, -12.25]**
Heterogeneity: Not applicable								
Test for overall effect: Z = 3.27 (P = 0.001)								

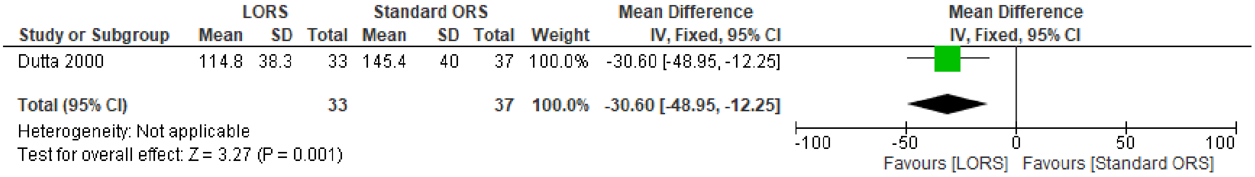

Study ID	D1	D2	D3	D4	D5	Overall
Dutta 2000	+	+	+	+	+	+
Sarker 2001	+	+	+	+	+	+

Figure S15: Forest Plot and ROB 2 for Stool Output (ml/kg/d)

Study or Subgroup	LORS Mean	LORS SD	LORS Total	Standard ORS Mean	Standard ORS SD	Standard ORS Total	Weight	Mean Difference IV, Fixed, 95% CI
Dutta 2000	61	24.5	33	75	29.4	37	100.0%	-14.00 [-26.63, -1.37]
**Total (95% CI)**			**33**			**37**	**100.0%**	**-14.00 [-26.63, -1.37]**

Heterogeneity: Not applicable
Test for overall effect: Z = 2.17 (P = 0.03)

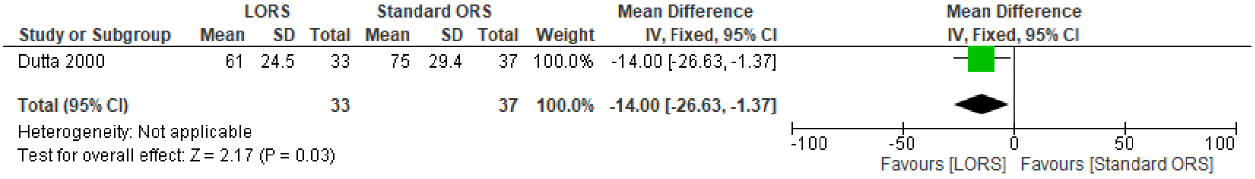

Study ID	D1	D2	D3	D4	D5	Overall
Dutta 2000	+	+	+	+	+	+
Sarker 2001	+	+	+	+	+	+

Figure S16: Forest Plot and ROB 2 for ORS Intake (ml/kg/d)

Study or Subgroup	LORS Mean	LORS SD	LORS Total	Standard ORS Mean	Standard ORS SD	Standard ORS Total	Weight	Mean Difference IV, Fixed, 95% CI
Dutta 2000	100	34.6	33	121.4	48.6	37	100.0%	-21.40 [-41.01, -1.79]
**Total (95% CI)**			**33**			**37**	**100.0%**	**-21.40 [-41.01, -1.79]**

Heterogeneity: Not applicable
Test for overall effect: Z = 2.14 (P = 0.03)

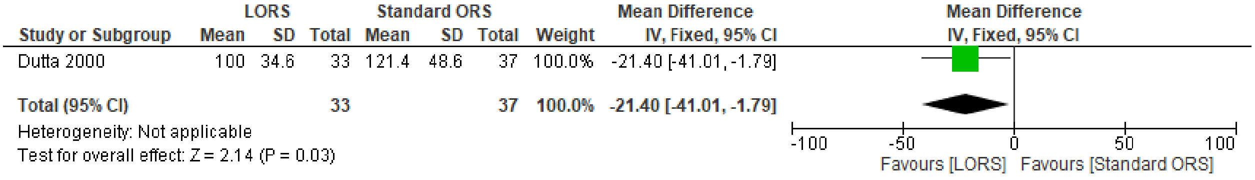

Study ID	D1	D2	D3	D4	D5	Overall
Dutta 2000	+	+	+	+	+	+
Sarker 2001	+	+	+	+	+	+
